# Development of 3D-Printed Gel-Based Supplement-Containing Tablets with Tailored Release Profiles for Neurological Pain Management

**DOI:** 10.3390/pharmaceutics17091168

**Published:** 2025-09-06

**Authors:** Jurga Andreja Kazlauskaite, Inga Matulyte, Jurga Bernatoniene

**Affiliations:** 1 Department of Drug Technology and Social Pharmacy, Lithuanian University of Health Sciences, LT-50161 Kaunas, Lithuania; jurga.andreja.kazlauskaite@lsmu.lt (J.A.K.); inga.matulyte@lsmu.lt (I.M.); 2 Institute of Pharmaceutical Technologies, Lithuanian University of Health Sciences, LT-50161 Kaunas, Lithuania

**Keywords:** semi-solid extrusion, SSE, three-dimensional printing, 3DP, neuropathy, pain, drug delivery, gel-based

## Abstract

**Background/Objectives**: Neuropathic pain, resulting from damage or pathology affecting the somatosensory nervous system, is a prevalent form of chronic pain that significantly impacts quality of life. Combined therapies are often utilised to manage this condition. Three-dimensional printing (3DP) offers a promising approach for personalising medication doses and dosage forms to meet individual patient needs. **Methods**: In this study, a formulation suitable for 3D printing was developed using magnesium citrate, uridine monophosphate, vitamins B_3_ (niacin), B_6_ (pyridoxine), B_12_ (cobalamin), B_9_ (folic acid), and spermidine to create a novel gel-based oral tablet for the targeted treatment of neurological pain. The antioxidant potential of the active pharmaceutical ingredients (APIs) was assessed using the 2,2-diphenyl-1-picrylhydrazyl (DPPH) and 2,2′-azino-bis(3-ethylbenzothiazoline-6-sulfonic acid) (ABTS) methods. The physical properties of the tablets were evaluated using a texture analyser, while the in vitro release profiles were determined by high-performance liquid chromatography (HPLC). **Results**: Results demonstrated that pectin–gelatin tablets hardened over time, with higher citric acid concentrations further enhancing this effect. Formulation AVII exhibited good hardness and low stickiness. Formulation AV, however, showed poor performance across all physical parameters and lacked sufficient structural integrity for practical application. While uridine monophosphate, B_12_, and B_9_ showed no significant differences in the release profiles of the tablets, spermidine, B_6_, and B_3_ displayed statistically significant variations. Specifically, AVII outperformed AV in terms of spermidine and B_6_ release, and AV showed a higher release of B_3_ compared to AV. **Conclusions**: The AVII tablet demonstrates potential for use in combined therapy targeting neurological pain disorders.

## 1. Introduction

Neuropathic pain, caused by trauma, surgical intervention, or disease of the somatosensory system, is a highly debilitating condition with unmet clinical needs [1]. It covers a wide range of peripheral and central disorders, including diabetic neuropathy, herniated disc, spinal cord injuries, nerve injuries, trigeminal neuralgia, herpes zoster, multiple sclerosis, and stroke [2,3]. Despite ongoing efforts, neuropathic pain remains challenging to manage due to the limited efficacy of conventional treatments and the need for more personalised, phenotype-based therapeutic approaches [4].

Many vitamins, minerals, and natural substances have been explored as supplements to help manage neurological pain. While they might not directly relieve pain, they can support nerve health, reduce inflammation, or enhance the effectiveness of pain medications [5].

Magnesium lacks direct analgesic properties but exerts antinociceptive effects through antagonism of N-methyl-D-aspartate (NMDA) receptors, thereby inhibiting calcium influx. Although this mechanism indicates therapeutic potential for neuropathic pain, clinical findings remain mixed [6,7].

A clinical study involving patients with peripheral neuropathy demonstrated that supplementation with uridine monophosphate, folic acid (B_9_) and cobalamin (B_12_) significantly reduced neuropathic pain intensity and permitted decreased reliance on analgesic medications, thereby decreasing the intensity of side effects [4]. Multiple studies have shown that B group vitamins, particularly B_1_, B_3_, B_6_, and B_12_, are beneficial in neuropathy management due to their supportive role in nervous system function [8]. Furthermore, C. G. Jolivalt et al. reported synergistic nerve-regenerating effects when combining vitamins B_1_, B_6_, and B_12_ in diabetic rat models [9].

The use of spermidine in a rat model of chronic constriction injury has shown potential to reduce neuropathic pain by lowering oxidative stress, improving tissue health, and modulating pain-related behaviour [10].

Although each of these compounds—magnesium, B-group vitamins, uridine, and spermidine—has demonstrated individual therapeutic benefits, their combined use may produce additive or synergistic effects through complementary mechanisms. Magnesium reduces excitotoxicity via NMDA receptor antagonism, creating a neuroprotective environment [11]. B vitamins and uridine support nerve regeneration, neurotransmitter synthesis, and membrane repair [12]. Spermidine contributes antioxidant and anti-inflammatory effects via autophagy regulation [13]. Together, these agents may address multiple pathological aspects of neuropathic pain simultaneously—such as neuronal damage, inflammation, and impaired regeneration—thus enhancing overall therapeutic efficacy beyond what each compound could achieve alone [14]. Novel formulation strategies are required to optimise the therapeutic potential of neuroactive and supportive compounds, especially those that ensure precise dosing and tailored drug release.

Three-dimensional printing (3DP) offers significant potential and enables the fabrication of personalised oral dosage forms with controlled release characteristics, precise geometry, and combination therapy capacity. Various 3DP techniques have been employed in pharmaceutical research and development, including fused deposition modelling (FDM), semi-solid extrusion (SSE), inkjet printing, binder jetting, and stereolithography (SLA) [15]. Among these, FDM involves the deposition of melted thermoplastic polymers and has been successfully used to create polypills and sustained-release tablets, although its high processing temperatures limit the use of thermolabile drugs [16]. SSE, by contrast, operates at lower temperatures and is better suited for incorporating heat-sensitive ingredients such as vitamins, amino acids, and biologics. It uses gel- or paste-like materials and allows direct extrusion into chewable tablets, orodispersible films, or flexible formulations for specific patient groups [17,18].

In particular, SSE has demonstrated strong potential for personalised medicine applications, such as in the management of neuropathic pain, where patient needs vary in terms of symptom profile, comorbidities, and swallowing ability [18].

Three-dimensional printing (3DP) technology in pharmaceuticals enables the production of custom-made, personalised medication [19]. Among the various 3DP techniques, semi-solid extrusion (SSE) has shown particular promise for pharmaceutical applications, especially when working with gel-based matrices and tablets. This technique allows the direct printing of gel formulations at low temperatures, preserving the integrity of heat-sensitive compounds [20]. Chewable gel tablets improve patient comfort and autonomy, especially in home-care settings. They do not require water for administration and are easy to carry and consume [21,22]. These tablet formulations present a promising approach for managing neurological pain, especially in patients with dysphagia and elderly individuals who have difficulty swallowing or handling traditional oral medications. These personalised gels can ensure controlled drug release and improved patient compliance [20,23].

This study aims to develop a novel gel-based oral tablet formulated using 3D SSE printing technology and to evaluate its quality and active ingredient release characteristics.

## 2. Materials and Methods

### 2.1. Materials

Gelatin (Carl Roth GmbH and Co. KG, Karlsruhe, Germany), apple pectin (Golden Peanut Gourmet, Egestorf, Germany), and distilled water (LUHS laboratory, Kaunas, Lithuania), sugar (Panevezio cukrus, Panevezys, Lithuania), citric acid (Carl Roth GmbH and Co. KG, Karlsruhe, Germany) were used as a chewable gel tablet basis. Magnesium citrate, uridine monophosphate, B3, B6, folic acid, spermidine, B12, and maltodextrin were brought from Carl Roth GmbH and Co. KG, Karlsruhe, Germany.

Based on the ingredient information provided in the “safety data sheets”, the decomposition temperatures of each substance are: Magnesium citrate begins to decompose at temperatures exceeding 200 °C. Uridine monophosphate decomposes at around 202 °C, vitamin B3 at 238 °C, vitamin B6 at 85 °C, and folic acid at 105 °C. Spermidine does not have a specific decomposition temperature like typical chemical compounds; however, it can degrade with prolonged exposure to elevated temperatures, with a boiling point between 128 and 130 °C. Vitamin B12 decomposes within the temperature range of 135–149 °C.

Purified water was prepared using a GFL2004 system (GFL, Burgwedel, Germany). HPLC-grade and analytical-grade reagents were used: hydrochloric acid, sodium hydroxide, acetic acid, methanol, and acetonitrile (Sigma Aldrich, Hamburg, Germany). The 96% ethanol was obtained from Vilniaus Degtinė (Vilnius, Lithuania). 2,2′-azino-bis(3-ethylbenzothiazoline-6-sulfonic acid) (ABTS) and acetic acid obtained from Sigma-Aldrich (Buchs, Switzerland). 2,2-diphenyl-1-picrylhydrazyl radical (DPPH) (BBL, Baltimore, MD, USA) were also utilised. Folin–Ciocalteu’s phenol reagent (Merck, Darmstadt, Germany).

### 2.2. Powders Physical Properties Evaluation

#### 2.2.1. Powder Flow

The compressibility index and the Hausner ratio were performed according to the Ph. Eur. 11.5 chapter 2.9.36 [24]. The values were calculated from a powder’s untapped and tapped bulk volume. Compressibility index (per cent) 1–10, Hausner ratio 1.00–1.11 excellent; compressibility index 11–15, Hausner ratio 1.12–1.18 good; compressibility index 16–20, Hausner ratio 1.19–1.25 fair; compressibility index 21–25, Hausner ratio 1.26–1.34 passable; compressibility index 26–31, Hausner ratio 1.35–1.45 poor; compressibility index 32–37, Hausner ratio 1.46–1.59 very poor; compressibility index > 38, Hausner ratio > 1.60 very, very poor.

#### 2.2.2. Moisture of the Powder

The powders’ moisture content was assessed using an MLB apparatus (KERN & Sohn GmbH, Balingen, Germany). The measurements were made three times and expressed as mean ± standard deviation (S.D.).

### 2.3. Three-Dimensional Printing

#### 2.3.1. Three-Dimensional Gel Tablets’ Base Preparation

The formulations are provided in Table 1. The optimal procedure for preparing the base involved precisely weighing the required amount of gelatin, followed by its hydration in water. The hydrated gelatin was allowed to swell for 20 min to ensure full absorption of water. Afterwards, the swelled gelatin was subjected to mixing at a controlled temperature of 85 °C using an IKA Eurostar mixer to facilitate uniform dispersion.

Pectin, along with an additional 5 g of water, was then incorporated into the gelatin mixture to enhance its viscosity and structural properties. Subsequently, sugar and citric acid were added to the blend. The mixture was continuously stirred and maintained at the elevated temperature until a homogeneous paste of consistent texture and composition was achieved.

The resulting paste was combined with active components (magnesium citrate, uridine monophosphate, B3, B6, folic acid, spermidine, B12, and maltodextrin) and maintained at 60 °C in a water bath. The 3D apparatus’s syringe was also heated to 60 °C to ensure proper extrusion. The formulation with APIs has an ‘A’ before the base number.

#### 2.3.2. Three-Dimensional Tablet Printing Parameters

The 3D printing was performed using the M3DIMAKER 1, a pharmaceutical-grade single printhead 3D printer developed by FabRx (London, UK). This semi-solid extrusion (SSE) printer was configured for printing at temperatures ranging from 40 to 60 °C using syringe-loaded gel-based formulations. The printer’s operation was controlled via dedicated software that allowed precise adjustment of layer geometry, flow rate, and deposition parameters.

The finalised printing conditions were as follows: nozzle diameter: 0.6 mm; tablet dimensions: 15 mm × 15 mm × 6 mm; layer thickness: 0.4 mm; printing pattern: rectilinear; feed rate: 60; infill density: 18%. The tablet dimensions were selected based on preliminary experiments in which different sizes were assessed for patient acceptability and orodispersibility. These dimensions ensured tablets that were easy to handle and rapidly dispersible in the oral cavity. The nozzle head diameters ranged from 0.6 mm to 1.2 mm.

The other printing parameters were optimised through iterative testing to balance mechanical integrity, printing resolution, and visual uniformity. Specifically, a 0.4 mm layer thickness and 18% infill provided stable structures without compromising disintegration. The flow rate was adjusted between formulations to account for differences in rheology and ensure smooth, uninterrupted extrusion. This empirical optimisation ensured that tablets were printed with uniform structure, minimal deformation, and reproducible dimensions.

### 2.4. Texture Analysis and Stability of the Chewable Gel Tablets

The texture properties of chewable gel tablets, including firmness, springiness, hardness, and stickiness, were assessed using a texture analyser (Ta.XT.plus, Texture Technologies, New York, NY, USA) (n = 3).

For firmness and springiness, the following parameters were employed: return speed of 10 mm/s, applied force of 1 g, 50% strain, pre-test and test speeds of 1.00 mm/s, post-test speed of 10 mm/s, hold time of 60 s and trigger force set at 5.0 g.

For hardness and stickiness, a probe of 20 mm height was used. The test speed was 2 mm/s, and a 5 mm distance was maintained between the probe and the tablet surface, where contact was made and the distance measured.

### 2.5. Antioxidant Activity and Quantification of Active Compounds in the Tablet

#### 2.5.1. Antioxidant Activity of the Active Compounds and Tablets

Antioxidant activity was assessed using the ABTS and DPPH methods, following the protocols previously performed by Kazlauskaite et al. [25].

The aqueous ABTS solution (7 mM) was mixed with potassium persulfate (2.45 mM), and the absorbance was measured at 650 nm. A trolox calibration curve (0–0.5 mg/g; y = 0.0001728x; R^2^ = 0.9832) was prepared, and results were expressed as milligrams trolox equivalent per gram dry weight (TE/g dw).

DPPH solution (0.1 mM in ethanol) was mixed with the sample. The reaction mixture was shaken and incubated in the dark at room temperature for 30 min. The absorbance was then measured at 517 nm, compared to the blank, using a spectrophotometer. A trolox calibration curve (0–0.016 mg/g; y = 0.00623x; R^2^ = 0.9923) was prepared, and results were expressed as milligrams trolox equivalent per gram dry weight (TE/g dw).

#### 2.5.2. Active Compounds’ Release In Vitro

The dissolution test for solid dosage forms was performed according to the European Pharmacopoeia 11.5, chapter 2.9.3 [26]. A tablet was placed in the basket, which was placed in a container with 500 mL of medium (temperature 36.6 °C); the basket rotation speed was 50 times per minute. Samples were taken after 15, 30, and 60 min. Sodium phosphate was added to alkalise the medium to the required pH value, and samples were taken after 75, 90, and 120 min. in the intestinal medium (time from the beginning of the tablet insertion). Samples are taken at the specified time interval, and the total amount of phenolic compounds is determined.

Gastric medium: 250 mL of 0.2 M sodium chloride solution is poured into a 1000 mL flask, 207 mL of 0.2 M hydrochloric acid is added and diluted to 1 L. pH = 1.5. Intestinal medium: the previous medium is alkalised with Na_3_PO_4_ × 12H_2_O (pH = 6.8).

The total phenolic content method was performed as described in our previous studies. Folin–Ciocalteu’s phenol reagent and 7% (*w*/*v*) sodium carbonate were employed for the reaction. The absorbance was measured after one hour using a spectrophotometer (765 nm) (Shimadzu UV-1800, Kyoto, Japan). The calibration curve used gallic acid (0–0.1 mg/g; y = 11.108; R^2^ = 0.9981). The results were reported as gallic acid equivalent per gram dry weight (mg GA/g dw).

#### 2.5.3. Quantification of Active Compounds via HPLC

The analysis used a Waters Acquity H-Class liquid chromatograph with an Xevo TQD tandem mass spectrometer for detection. Chromatographic separation was achieved using a Waters BEH Amide column (150 mm × 2.1 mm, 1.7 µm particle size), which ensured high-resolution separation of the target compounds.

The prepared sample was diluted 20-fold with the appropriate solvent before analysis to achieve the desired concentration range.

A gradient elution method was employed: 5% A/95% B at 0 to 1 min; 30% A/70% B at 1 to 3.9 min; 70% A/30% B at 3.9 to 6.4 min; 5% A/95% B at 6.4 to 10 min. The flow rate was 1 mL/min, and the injection volume was 10 μL. The column temperature was maintained at a constant 40 °C to ensure stable retention and reproducibility of the separation. Eluent A: 10 mmol ammonium formate solution in water containing 0.125% sulfonic acid. Eluent B: Acetonitrile.

The analysis utilised positive and negative electrospray ionisation (ESI) modes to accommodate a broad range of analytes. The capillary voltage was set at 3500 V, and the ion source temperature was maintained at 120 °C. Nitrogen gas was used for desolvation at a flow rate of 650 L/h to ensure efficient ionisation.

The analysis of the target analytes was performed in multiple reaction monitoring (MRM) mode, enabling high sensitivity and specificity. Two specific fragmentation channels were monitored for each analyte: the fragment with the highest intensity was used for quantitative evaluation, while the fragment with lower intensity was employed to confirm analyte identity. This dual-channel approach ensured both accurate quantification and reliable confirmation of the analytes. Comprehensive method validation results are available in the Supplementary Data.

### 2.6. Statistical Analysis

Data were analysed using IBM SPSS Statistics version 20.0 and Microsoft Office Excel. All experiments were performed at least three times, and results were expressed as mean values ± standard deviation (SD), where applicable. The Kruskal–Wallis one-way ANOVA test with multiple comparisons was used to assess whether the differences between the samples were statistically significant (*p* < 0.05).

## 3. Results and Discussion

### 3.1. The Quality of Active Ingredients

The Hausner Ratio (HR) and Compressibility Index (Carr’s Index, CI) are indicators of powder flowability. Lower values correspond to better flowability, according to the pharmacopoeia [24].

Magnesium citrate has HR ≈ 1.18 and CI ≈ 15.4%, which falls in the good flow range (Table 2). This suggests magnesium citrate is a free-flowing powder with minimal interparticle friction. Uridine monophosphate and pyridoxine (HR ~1.30, CI ~23%) fall within a fair to passable flow range. In contrast, niacin (HR 1.353, CI 26.1%) and cobalamin (HR ~1.33, CI 25%) exhibit poor flowability. A Carr’s Index above 25% is associated with poor flowability; these values imply that powders are more cohesive and may not flow smoothly [27]. The poorest values were observed for folic acid with HR ~1.67 and CI 40%—extremely poor flowability.

Moisture content is another critical factor for powder excipients and APIS, affecting both their processing behaviour and the stability of the formulation. Even a few percent of moisture can significantly alter powder properties [28].

Uridine monophosphate had a moisture content of 16.11%, indicating it is quite hygroscopic (Table 2). Such a high moisture level will make the powder noticeably damp or prone to agglomeration. It may form lumps during handling and not flow freely without agitation [29]. Folic acid (7.75% moisture) also has a relatively high moisture level, which can contribute to its already poor flow by promoting particle cohesion. In contrast, magnesium citrate, pyridoxine, and niacin have low moisture content (~2–3.5%), and are less likely to clump due to humidity.

### 3.2. Antioxidant Activity of Active Ingredients

The antioxidant activity of the used APIs was determined using ABTS and DPPH radical scavenging methods (Table 3). Pyridoxine (vitamin B6) exhibits the highest antioxidant activity, with an ABTS value of 345.64 mg TE/g and a DPPH value of 7.21 mg TE/g, making it the most potent radical scavenger in this study.

Folic acid also demonstrated relatively high antioxidant activity. Magnesium citrate, niacin, spermidine, and cobalamin (vitamin B12) exhibited moderate antioxidant potential, with ABTS values ranging from 59.86 to 70.29 mg TE/g and DPPH values of approximately 4.15 to 4.26 mg TE/g. Uridine monophosphate has the lowest ABTS value (62.08 mg TE/g), suggesting a comparatively weaker antioxidant effect, but its DPPH activity (4.09 mg TE/g) remains similar to the others, indicating some free radical scavenging ability. These compounds are known for their roles in nerve function, neurotransmitter synthesis, and cellular energy metabolism, all of which are critical in managing neurological conditions [30,31]. The significantly higher values of pyridoxine highlight its strong potential in combating oxidative damage. In contrast, the moderate activity of the other compounds suggests they also play a role in maintaining redox balance.

The active ingredients’ antioxidant properties are crucial in mitigating oxidative damage, a key factor in the progression of chronic neuropathic pain and neurodegenerative diseases [32]. Magnesium citrate, known for its muscle-relaxing and nerve-stabilising effects, has also been incorporated into treatments for migraines and nerve pain, while spermidine has gained attention for its role in neuronal regeneration and synaptic plasticity [31,33,34,35]. The synergistic effects between compounds enhance their individual neuroprotective effects, support nerve repair mechanisms, reduce neuroinflammation, and stabilise nerve function, making them valuable components in managing neurological pain disorders [30].

### 3.3. SEE 3D Printing of the Tablets and the Analysis of the Physical Parameters

Different formulations of tablet bases were prepared using gelatin and pectin as the gelling agents, with variation in their concentrations. The compositions were further modified by adjusting the sugar and citric acid levels in the base matrix. The gelation of pectin occurs primarily in the presence of sufficient sugar and an acidic pH. In the current formulations, citric acid reduces the pH, promoting the gelation of pectin and enhancing the network’s firmness and stability [36]. Gelatin, on the other hand, undergoes thermoreversible gelation: it forms a gel upon cooling due to the partial reformation of triple helices from denatured collagen chains [37]. The combination of gelatin and pectin allows the development of a dual-gelling system, where gelatin provides elasticity and melt-in-mouth texture, while pectin contributes to the structural integrity and stability of the formulation [36,37].

#### 3.3.1. Three-Dimensional Printing Parameters

Initially, the printing parameters were optimised using base formulations without the active pharmaceutical ingredient (API). This was performed to evaluate the rheological and mechanical properties of the gelatin–pectin matrices.

**Figure 1:**
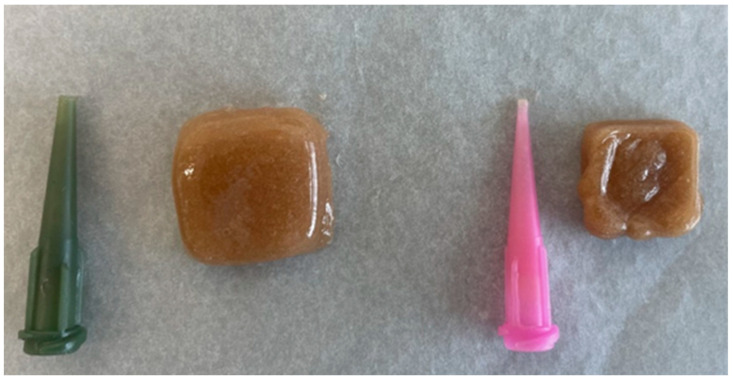
Printing needles and the resulting tablets when printing with them.

Different nozzle sizes and printing speeds were tested to determine the optimal conditions for achieving precise shape fidelity and structural stability during semi-solid extrusion-based 3D printing (SSE). The choice of nozzle diameter and print speed directly influenced the extrusion flow rate, line thickness, and resolution of the printed structures, which are critical factors for successfully fabricating uniform and reproducible dosage forms (Figure 1). The needles used in our study varied in size, with diameters ranging from 0.6 mm to 1.28 mm. The measurements correspond to a range of gauge sizes from approximately 30 G to 18 G.

**Figure 2:**
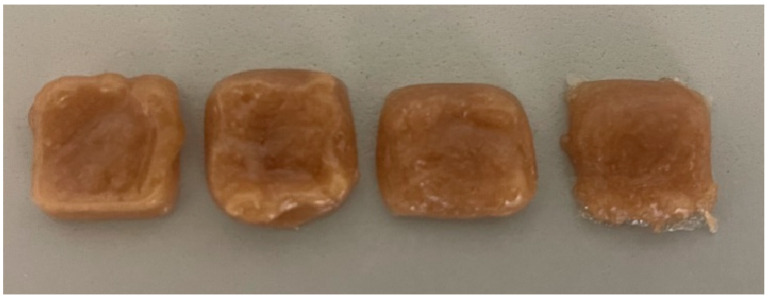
Tablets at flow rates ranging from 60 to 100.

**Figure 3:**
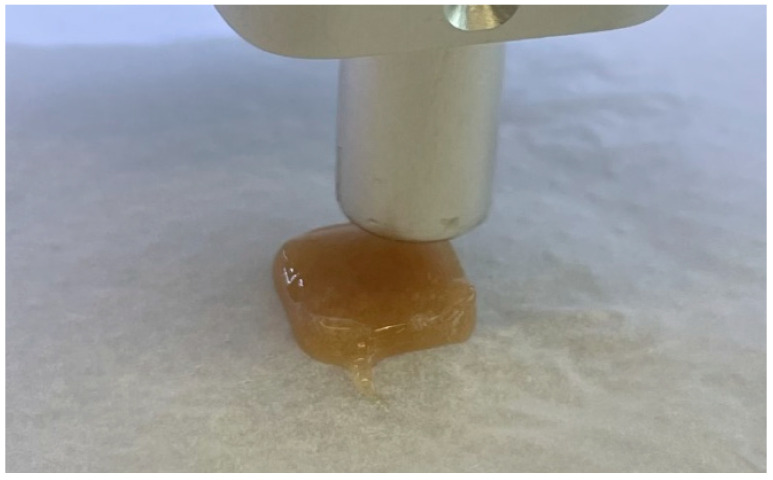
Final tablet with selected parameters.

The appearance of tablets depended on printing speed, tablet size, and the type of printing needle used (see Figure 1, Figure 2 and Figure 3). The acidity of active compounds influenced the curing speed. In this formulation, an infill density of at least 15% should be used, as acids that promote pectin solidification can clog the needle of the apparatus. Adjustments were made to the printing needles because of the **rapid curing when using smaller-diameter needles (0.6 mm)** and **overflow** when using larger-diameter needles (1.28 mm) (Figure 1). Using a larger nozzle diameter (**greater than 0.8 mm**) made it impossible to produce smaller tablets, as this resulted in over-extrusion during printing. This caused the tablets to exceed their intended boundaries and deform over time, with layers spreading laterally. Although the tablets appeared intact when freshly printed, they began to sag at the edges after a short period (Figure 1). Metal needles caused clogging and were therefore excluded from further use.

A 0.6 mm nozzle produces finer and more detailed tablets, although imperfections remain on the base and tablet surface (Figure 2). As mentioned before, using a smaller needle resulted in faster tablet curing, so there was a need to change the parameters. Changing the printing speed may alter the tablet’s appearance, even if other parameters remain constant (Figure 2). A slow feed rate and flow rate ensures precise layering, yet adjustments are necessary to prevent blockages. The flow rate was increased to allow the material to flow faster and to avoid clogging.

By refining these parameters on the API-free bases, a standardised printing protocol was established before incorporating the active compound into the formulations.

Appropriate parameters yield the final tablet formulation (see Figure 3). The optimised settings (18% infill, 0.4 mm layer thickness) were found to provide the best balance between appearance and performance. Differences in formulation viscosity necessitated individual flow rate adjustments to maintain consistent extrusion quality. These parameter refinements ensured that all printed tablets maintained uniform geometry, acceptable mechanical stability, and visual uniformity suitable for patient use.

#### 3.3.2. The Analysis of the Tablets’ Physical Parameters

**Figure 4:**
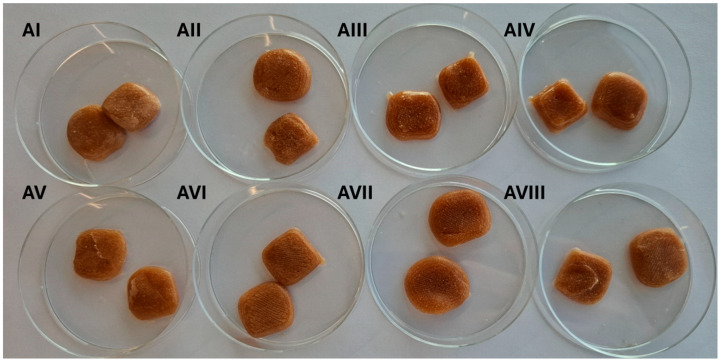
3D-Printed Tablets with APIs AI–AVIII — formulation differences provided in Table 1.

The mechanical properties of tablets depend on the ingredients used and their concentration. A total of sixteen formulations, eight without active pharmaceutical ingredients (APIs) (I-VIII) and eight containing APIs (AI-AVIII) (Figure 4), were evaluated for their physical properties: firmness, springiness, hardness, and stickiness (Table 4). Each sample was assessed immediately after preparation and again after a two-week storage period to determine mechanical stability and suitability for use as a chewable 3D-printed dosage form.

Citric acid can influence the gel structure and stability by modulating pH and potentially inducing crosslinking effects with pectin. As a result, base formulations with 1% citric acid became excessively hard (the texture analyser could not record a value, as it exceeded 6500 g) after two weeks, whereas formulations with 0.5% citric acid remained measurable and usable.

Variations in the concentrations of gelatin, pectin, sugar, and citric acid significantly influenced the mechanical properties of the 3D-printed formulations. Although a higher citric acid concentration would typically weaken gelatin-based gels, the obtained gels were still quite hard [38,39,40].

Increased levels of gelatin and pectin generally enhanced firmness and springiness; however, when combined with higher citric acid (1%), several formulations became excessively hard after storage, rendering them unsuitable as chewable dosage forms. Conversely, formulations with moderate gelatin and pectin levels, lower citric acid (0.5%), and higher sugar content (30%) maintained a desirable balance of firmness, elasticity, and low stickiness over time (Table 4, Figure 4).

Formulations AI–AVIII (Figure 4), which included APIs, generally exhibited slightly altered mechanical profiles due to the presence of active substances. In the initial evaluation, Formulation AI ranked highest among the API-containing samples, followed by AII and AIII. These formulations exhibited high mechanical strength and low stickiness, making them suitable for 3D printing and handling. The information on how used APIs could influence the gel texture is very limited. Only information about the use of maltodextrin is available. The lox concentrations of this compound can enhance gel firmness due to its ability to increase the total solids content, leading to a denser gel network [41].

After two weeks, a significant differentiation in texture was observed. Several formulations, particularly AI, AII, and AIII, became too hard to measure. As such, these formulations were considered unfavourable. Among the API-containing formulations, only AV, AVI, and AVII remained within measurable and chewable ranges after two weeks. Of these, Formulation AVI demonstrated the most balanced and desirable profile, maintaining high firmness, strong elasticity, and low stickiness, while avoiding excessive hardness. Formulation AVII also retained good hardness and low stickiness, but its lower springiness reduced its overall suitability. Formulation AV exhibited poor performance across all physical parameters and lacked sufficient structural integrity for practical application.

### 3.4. In Vitro Release of the Selected 3D Tablets

Three tablets, chosen based on their texture, were selected to analyse their in vitro release—AV, AVI, and AVII. The release of uridine monophosphate, spermidine; B12; B6; B3; B9 was measured.

**Figure 5:**
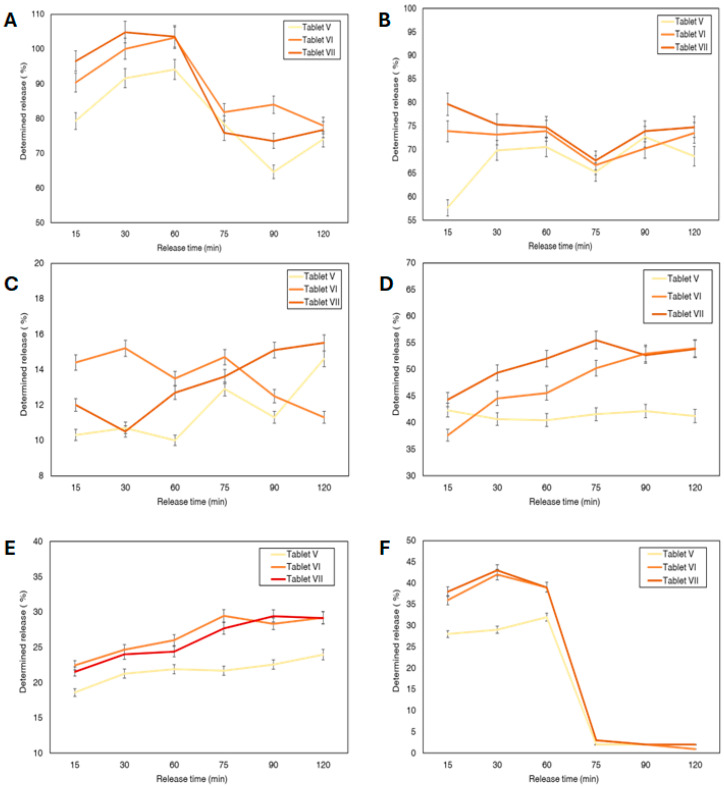
In vitro release from V, VI and VII formulations. (**A**)—uridine monophosphate, (**B**)—spermidine; (**C**)—B_12_; (**D**)—B_6_; (**E**)—B_3_; (**F**)—B_9_.

For uridine monophosphate, the ANOVA test revealed no statistically significant difference in the release profiles between the three formulations, with a *p*-value of 0.3761. This indicates that all three tablets (AV, AVI, and AVII) released uridine monophosphate at similar rates, meaning there was no significant advantage of one formulation over the others regarding the release of this API (Figure 5A). The highest concentration of uridine monophosphate was released in the stomach and it gradually decreased.

For spermidine, the ANOVA test showed a statistically significant difference between the formulations, with a *p*-value of 0.0352. This suggests that at least one tablet formulation had a significantly different release profile. AVII released spermidine at a significantly higher rate than AV. However, no significant difference was found between tablet AVI and AVII, nor between AV and AVI. Therefore, AVII released the highest percentage of the spermidine (Figure 5B). The spermidine release was similar in the stomach and the intestinal juice. Changing the media, the concentration of spermidine in intestinal juice dropped, but after 90 min, the release concentration increased.

Similar results were observed with the release of B3 (Figure 5E). Sample AVI had a significantly higher B3 release rate than AV, while Tablet V showed a significantly higher release of B3 compared to Tablet AVII. Most of B3 was released in the intestinal media, In human body it is absorbed including both stomach and small intestine, therefore the release in either is generally effective [42].

There was no significant difference in the release profiles among the three formulations for B_9_ and B_12_ (Figure 5C,F). For B_6_, Tablet AVII released B_6_ at a significantly higher rate than AV, but no significant difference was found between AVI and AVII (Figure 5D). B_9_ was mainly released in the stomach media while most of B_6_ and B_12_ were released in the intestinal media. A slight decrease in percentage release was observed in some cases during extended dissolution (vitamin B9 in Figure 5F). This trend was reproducible across repeated experiments, suggesting it is not the result of random variability. While the release should ideally plateau under standard dissolution conditions, this consistent decline may reflect compound–matrix interactions, instability under changing pH, or measurement limitations. However, as no direct evidence of degradation or adsorption was obtained in this study. Further investigation is needed to clarify this behaviour. In particular, the early release of B_9_ in the gastric medium may lead to degradation and reduced bioavailability, indicating that formulation improvements are needed to shift its release to the intestinal phase. The B_6_ and B_12_ can show early release to enable later intestinal absorption [43,44,45].

The differences in release percentages among the active compounds (Figure 5A–F) appear to result largely from their individual physicochemical properties, including solubility, stability, and how they interact with the tablet matrix. For example, uridine monophosphate showed rapid and complete release—occasionally exceeding 100%—which is likely due to its high solubility in water and ease of diffusion through the matrix. Values slightly above 100% may also reflect minor analytical variability or enhanced solubility from excipients in the formulation. On the other hand, compounds like vitamin B_12_ and vitamin B_6_ released more slowly and to a lesser extent, which could be attributed to their lower solubility or stronger retention within the semi-solid structure [46]. Vitamin B_9_ showed a distinct release pattern, with an initial burst followed by a rapid decline, possibly due to degradation or precipitation in the medium. Overall, even though all formulations were printed under the same conditions, the release behaviour was clearly influenced by the specific characteristics of each compound.

## 4. Conclusions

The resulting printable paste was combined with active components including magnesium citrate, uridine monophosphate, vitamin B3 (niacin), vitamin B6 (pyridoxine), folic acid (vitamin B9), spermidine, and vitamin B12 (cobalamin). These compounds were selected based on prior evidence of their antioxidant activity, which was confirmed by our results. The 3D SSE-printed tablets showed good structural integrity, with formulation AI demonstrating the best performance in terms of strength and low stickiness, followed by AII and AIII, supporting their suitability for personalised oral delivery.

Despite limited data on how individual APIs affect gel texture, maltodextrin was identified as a key excipient capable of enhancing gel firmness at low concentrations by increasing the total solids content and contributing to a denser gel network. In vitro release studies showed that uridine monophosphate, B12, and B9 showed no significant differences in tablet release profiles; however, spermidine, B6, and B3 showed statistically significant differences. Specifically, sample AVII outperformed AV for both spermidine and B6, and AVI showed a higher release of B3 than AV. These findings show the importance of formulation design in modulating drug release and position AVII as the most effective formulation for the targeted delivery of neuroactive compounds. However, the early gastric release of B9 may compromise its stability and bioavailability, highlighting the need for further formulation refinement.

In the future, the influence of the amount of ingredients on the physical parameters of 3D tablets will be evaluated and, based on the experimental design, the most suitable formulation using these active ingredients will be modelled and evaluated. We recommend vacuum sealing the 3D-printed gel tablets to retain their soft texture and prevent excessive hardening during storage.

## Figures and Tables

**Table 1:** The chewable gel base formulation in grams.

		I	II	III	IV	V	VI	VII	VIII
	
Gelatin		9	9	10	10	9	9	10	10
Pectin		7	7	8	8	7	7	8	8
Sugar		28	30	28	30	28	30	28	30
Citric acid		1	1	1	1	0.5	0.5	0.5	0.5
Water		60	60	60	60	60	60	60	60

**Table 2:** The results of the powder’s HR, CI and moisture.

		Hausner Ratio	Compressibility Index	Moisture, %
	
Magnesium citrate		1.182 ± 0.05	15.38 ± 0.54	2.12 ± 0.24
Uridine monophosphate		1.300 ± 0.15	23.08 ± 0.70	16.11 ± 1.84
Niacin		1.353 ± 0.21	26.09 ± 0.32	3.54 ± 1.01
Pyridoxine		1.300 ± 0.04	23.08 ± 0.43	2.15 ± 0.68
Cobalamin		1.333 ± 0.07	25.00 ± 0.29	4.18 ± 0.63
Folic acid		1.667 ± 0.04	40.00 ± 0.63	7.75 ± 0.41

**Table 3:** Antioxidant activity of the used APIs.

		ABTS, mg TE/g	±SD	DPPH, mg TE/g	±SD
	
Magnesium citrate		70.29	0.47	4.26	0.03
Uridine monophosphate		62.08	3.54	4.09	0.03
Niacin		65.94	0.68	4.26	0.08
Pyridoxine		345.64	2.06	7.21	0.08
Cobalamin		59.86	2.53	4.15	0.04
Folic acid		76.18	0.80	4.41	0.04
Spermidine		68.16	1.45	4.16	0.09

**Table 4:** The texture analysis of prepared tablets with and without APIs.

Samples	Firmness *		Springiness *		Hardness *		Stickiness *	
	Prepared	After 2 Weeks	Prepared	After 2 Weeks	Prepared	After 2 Weeks	Prepared	After 2 Weeks
I	933.94 ± 49.74	-	33.31 ± 6.17	-	538.06 ± 10.62	-	−45.57 ± 4.26	-
AI	1094.34 ± 45.55	-	25.1 ± 1.65	-	1281.02 ± 22.66	-	−434.63 ± 1.7	-
II	465.94 ± 99.37	5829.82 ± 161.96	35.35 ± 6.6	61.39 ± 3.68	701.29 ± 14.34	-	−46.77 ± 2.56	−1.15 ± 0.04
AII	900.45 ± 5.97	-	21.27 ± 1.12	-	1228.78 ± 38.48	-	−406.63 ± 23.89	-
III	902.42 ± 44.34	-	46.22 ± 2.73	-	1041.68 ± 33.57	-	−68.35 ± 0.68	-
AIII	1021.29 ± 185.01	-	23.43 ± 2.22	-	1218.39 ± 35.76	-	−309.4 ± 9.18	-
IV	1012.16 ± 119.62	-	40.81 ± 6.17	-	976.92 ± 36.25	-	−76.51 ± 3.99	-
AIV	1092.17 ± 144.78	-	26.87 ± 0.73	-	1249.62 ± 39.56	-	−249.19 ± 2.6	-
V	1275.69 ± 71.75	-	37.91 ± 0.42	-	1617.94 ± 39.93	-	−129.02 ± 12.81	-
AV	289.58 ± 116.11	3280.68 ± 774.6	8.84 ± 0.6	35.54 ± 2.12	413.35 ± 72.03	5354.99 ± 350.98	−30.11 ± 5.71	−0.12 ± 0.07
VI	1036.4 ± 200.46	-	36.7 ± 2.82	-	1359.33 ± 35.1	-	−80.43 ± 6.11	-
AVI	388.48 ± 127.61	4962.15 ± 252.92	7.06 ± 0.63	37.91 ± 0.42	618.19 ± 245.13	5845.23 ± 209.88	−1.66 ± 0.03	−0.13 ± 0.07
VII	989.49 ± 61.49	-	38.47 ± 2.23	-	1352.21 ± 29.08	-	−88.56 ± 1.99	-
AVII	394.5 ±110.27	5087.64 ± 809.44	11.17 ± 0.46	36.27 ± 4.47	704.63 ± 66.82	6045.34 ± 4.56	−15.08 ± 6.26	−0.09 ± 0.05
VIII	2754.46 ± 152.66	-	48.53 ± 0.25	-	2283.46 ± 21.96	-	−31.14 ± 6.89	-
AVIII	290.22	-	10.19 ± 2.37	-	506.37 ± 136.65	-	−1.53 ±0.05	-

* “-“ indicates that the sample was too hard to examine, value higher than 6500 g.

## Data Availability

The data that support the findings of this study are available from the corresponding author upon reasonable request. Otherwise, a majority of the links can be found in the references.

## Supplementary Materials

The following supporting information can be downloaded at: https://www.mdpi.com/article/10.3390/pharmaceutics17091168/s1.

## Abbreviations

ABTS	2,2′-azino-bis(3-ethylbenzothiazoline-6-sulfonic acid
DPPH	2,2-diphenyl-1-picrylhydrazyl
3DP	Three-dimensional printing
HPLC	High-performance liquid chromatography
SEE	Semi-Solid Extrusion
FDM	Fused Deposition Modelling
API	Active pharmaceutical ingredient
