# Supplementary material for: Development of 3D-Printed Gel-Based Supplement-Containing Tablets with Tailored Release Profiles for Neurological Pain Management
Source: Pharmaceutics. 2025 Sep 6;17(9):1168. doi: 10.3390/pharmaceutics17091168 (PMC12472980; doi:10.3390/pharmaceutics17091168)
Supplement: Supplementary file 1 [file pharmaceutics-17-01168-s001.zip › pharmaceutics-3790998-supplementary.pdf]

Specificity

Specificity of the method was confirmed by analysis of blank and spiked samples, which showed no interfering peaks at the retention times of the analytes

Table S1. Retention times and MRM transitions of analytes for method specificity assessment.

Analyte	Retention Time (min)	Molecular ion	Quantifier ion	Qualifier ion
Uridine monophosphate	5.21	325	97	113
Spermidine	6.25	146	72	84
Vitamin B3	1.14	123	80	50
Vitamin B6	2.25	170	152	134
Vitamin B9	4.84	442	295	120
Vitamin B12	5.30	679	359	147

Linearity

Linearity was evaluated over the designated concentration range for each analyte. Calibration curves were constructed using no fewer than six concentration levels, and the corresponding correlation coefficients (R^2 values) were determined.

Table S2. Calibration data and linearity parameters for the analysed compounds.

Analyte	Concentration Range (µg/mL)	Regression Equation	R^2 Value
Uridine monophosphate	580 – 3480	y = 304.744x + 12548.2	0.9842
Spermidine	6.775 – 162.61	y = 131641x + 55153	0.9822
Vitamin B3	0.48 – 21.95	y = 228841x + 30904	0.9874
Vitamin B6	2.57 – 154.2	y = 996292x + 78820	0.997
Vitamin B9	0.169 – 4.06	y = 54294.1x + 283.22	0.9995
Vitamin B12	0.118 – 7.13	y = 1273.06x + 9.59	0.9974

Accuracy

Accuracy was evaluated by spiking known quantities of each analyte into the sample matrix at three concentration levels (low, medium, and high). Percent recoveries were calculated at each level to assess accuracy

Table S3. Percent recoveries of analytes from spiked sample matrices at low, medium, and high concentration levels.

Analyte	Low (50 %)	Medium (100 %)	High (150 %)
Uridine monophosphate	99.2%	100.3%	101.1%
Spermidine	96.8%	98.7%	97.9%
Vitamin B3	100.5%	101.2%	99.4%
Vitamin B6	97.6%	99.8%	98.9%
Vitamin B9	95.9%	97.4%	96.8%
Vitamin B12	98.3%	99.7%	101.0%

Precision

Precision was assessed by performing six replicate injections at a single concentration level, and the relative standard deviation (RSD) of the peak areas was calculated. Intermediate precision was further evaluated by repeating the procedure over three consecutive days

4.1 Repeatability

Table S4. Repeatability results (RSD, %) for analytes across three concentration levels.

Analyte	Low (50%)	Medium (100%)	High (150%)
Uridine monophosphate	1.31 %	0.94 %	0.8 %
Spermidine	1.66 %	1.15 %	1.01 %
Vitamin B3	1.01 %	0.87 %	0.72 %
Vitamin B6	1.48 %	1.03 %	0.97 %
Vitamin B9	1.89 %	1.32 %	1.25 %
Vitamin B12	1.9 %	1.46 %	1.34 %

4.2 Intermediate Precision

Table S5. Intermediate precision results (RSD, %) for analytes across three concentration levels.

Analyte	Low (50%)	Medium (100%)	High (150%)
Uridine monophosphate	2.23 %	1.66 %	1.58 %
Spermidine	2.56 %	1.94 %	1.83 %
Vitamin B3	1.87 %	1.42 %	1.34 %
Vitamin B6	2.11 %	1.61 %	1.56 %
Vitamin B9	2.79 %	2.13 %	1.97 %
Vitamin B12	2.85 %	2.20 %	2.09 %

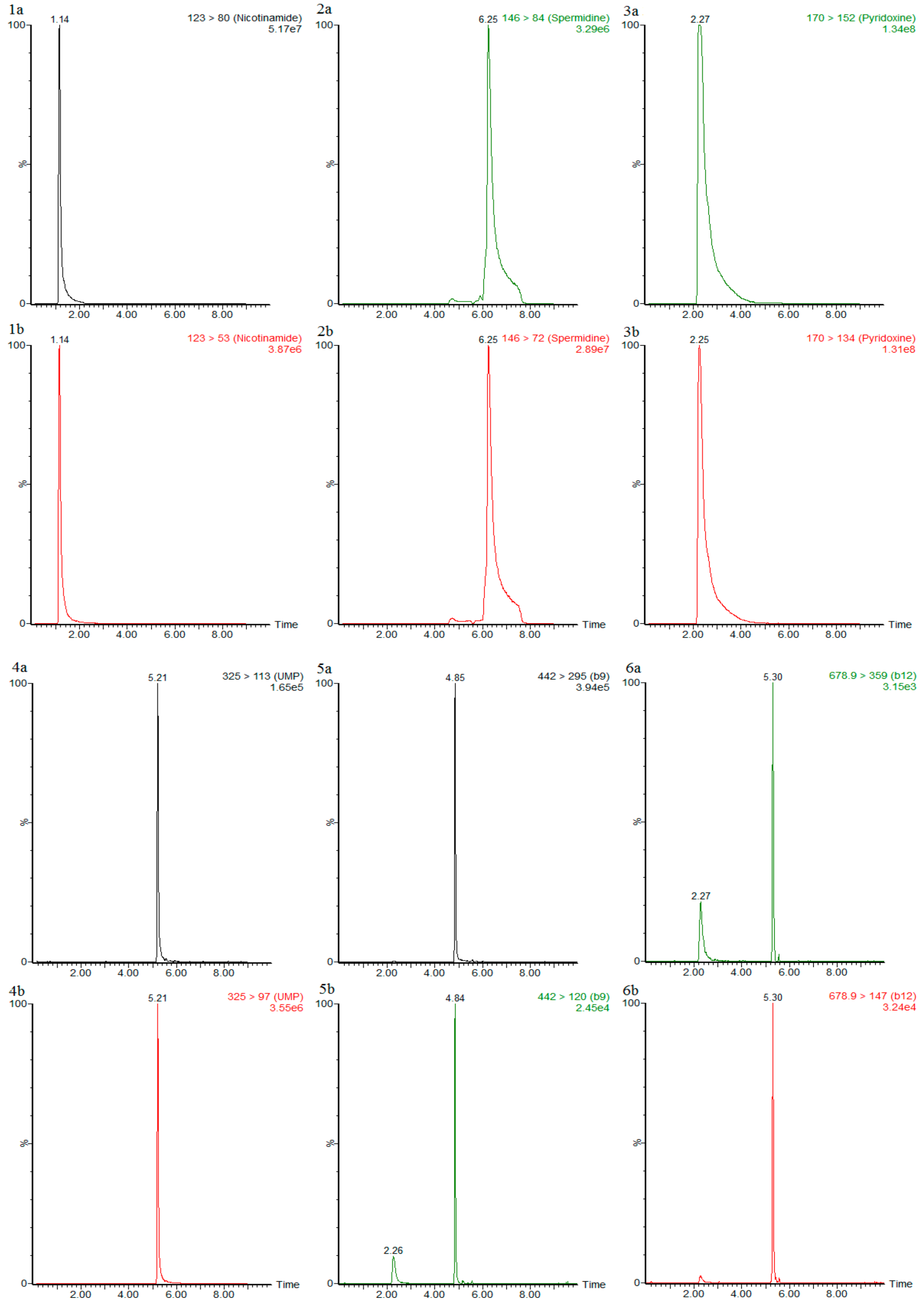

Figure S1. Selected MRM transition chromatograms of analytes. 1 – Vitamin B3, 2 – Spermidine, 3 – Vitamin B6, 4 – Uridine monophosphate, 5 – Vitamin B9, 6 – Vitamin B12. Letters a and b represent quantitative and qualitative MRM transitions.
